# Systematic Analysis of the Maize *CAD* Gene Family and Identification of an Elite Drought-Tolerant Haplotype of *ZmCAD6*

**DOI:** 10.3390/plants15020241

**Published:** 2026-01-13

**Authors:** Zhixiong Zhao, Wen Xu, Tao Qin, Jingtao Qu, Yuan Guan, Yingxiong Hu, Wenyu Xue, Yuan Lu, Hui Wang, Hongjian Zheng

**Affiliations:** 1 Shanghai Key Laboratory of Agricultural Genetics and Breeding, Shanghai Engineering Research Center of Specialty Maize, Crop Breeding and Cultivation Research Institution/CIMMYT-China Specialty Maize Research Center, Shanghai Academy of Agricultural Sciences, Shanghai 201403, China; 2 College of Life Sciences, Shanghai Normal University, Shanghai 201418, China

**Keywords:** maize, cinnamyl alcohol dehydrogenase (CAD), ZmCAD6, drought stress, haplotype analysis

## Abstract

Drought and salt stresses are major abiotic factors limiting maize yield. Lignin, a key cell wall component, plays a crucial role in boosting plant stress resistance. Cinnamyl alcohol dehydrogenase (CAD) is a vital enzyme at the late stage of lignin biosynthesis; however, a systematic study of its functions in abiotic stress responses and its potential for genetic improvement in maize remains lacking. In this study, we conducted the first comprehensive, multi-dimensional analysis of the maize *ZmCAD* gene family, including gene identification, evolutionary relationships, protein interaction networks, and stress-responsive expression patterns. We identified 9 *ZmCAD* members that showed significant functional divergence in evolution, structure, and expression patterns. Expression analysis revealed complex, tissue-specific responses of *ZmCAD* genes to drought and salt stress, with *ZmCAD6* strongly induced by drought. Importantly, through haplotype analysis of 157 waxy maize inbred lines, we successfully identified an elite haplotype (H3) of *ZmCAD6* that is significantly associated with improved drought tolerance in maize. This study not only clarifies the functional differentiation mechanisms of the *ZmCAD* gene family but also provides the identified elite *ZmCAD6-H3* haplotype as a valuable genetic resource and precise target for molecular breeding aimed at enhancing drought tolerance in maize.

## 1. Introduction

Maize, as a globally important food crop, faces severe challenges in arid, semi-arid, and salinized regions. Although its C4 photosynthetic pathway confers high water use efficiency, maize is highly sensitive to water stress during critical growth stages, and its root system is susceptible to changes in soil osmotic potential caused by salinity [1]. With the increasing global scarcity of agricultural water and the expansion of soil salinization, drought and salt stress severely constrain maize yield potential through synergistic mechanisms such as osmotic stress, ionic toxicity, and oxidative damage [2].

To cope with these adversities, plants have evolved complex defense mechanisms, among which, the strengthening and remodeling of the cell wall, particularly lignin deposition, has been confirmed as a core strategy for enhancing plant stress resistance [3,4]. Lignin accumulation not only reduces water loss and enhances mechanical strength to resist osmotic stress, but its synthesis process is also closely linked to signaling pathways such as reactive oxygen species (ROS) metabolism. In recent years, several studies have directly linked lignin synthesis to stress resistance in maize. For example, overexpression of *Zmhdz9* enhances drought tolerance by increasing abscisic acid (ABA) synthesis and lignin accumulation [5]; while the *ZmESBL* protein enhances the structural integrity of the Casparian strip in roots by regulating lignin deposition, thereby effectively controlling salt uptake and improving salt tolerance [6]. These findings highlight the critical role of the lignin metabolic pathway in maize stress resistance.

CAD catalyzes the final step in monolignol synthesis and is a key terminal enzyme in lignin biosynthesis [7]. In other major crops, the functional diversity of the *CAD* gene family has been extensively revealed. For instance, 47 *TaCAD* members have been identified in wheat, but only a subset has been confirmed as bona fide *CAD* enzymes [8]; *OsCAD6* in rice has been shown to participate in abiotic stress defense [9]; while a mutation in *SbCAD2* in sorghum directly leads to a significant reduction in lignin content [10]. Recent studies have identified a total of 90 CAD genes across six Brassica species, revealing that multiple members, particularly BnaCAD1 and BnaCAD6, are significantly differentially expressed under stress conditions such as drought and salinity [11]. Furthermore, the overexpression of CcCAD10 has been shown to enhance lignin accumulation in pigeon pea, thereby improving its salt tolerance [12]. Collectively, these studies indicate that the *CAD* gene family is typically a multi-member, functionally differentiated family that plays an important role in plant stress resistance.

However, compared to extensive research in other crops, a systematic investigation of the *CAD* gene family in maize, particularly its functional roles in abiotic stress responses and its potential for genetic improvement, remains a significant knowledge gap. To address this, the primary objective of this study was to perform a comprehensive, multi-dimensional analysis of the *ZmCAD* gene family. Specifically, we aimed to: (1) systematically identify *ZmCAD* members and elucidate their evolutionary relationships, gene structures, and protein interaction networks to infer functional divergence; (2) characterize the tissue-specific expression patterns of these genes under drought and salt stress to determine their distinct roles in stress adaptation; and (3) explore natural variations in *ZmCAD* genes through haplotype analysis using a panel of 157 waxy maize inbred lines. By integrating these analyses, this study sought to identify elite haplotypes that can serve as valuable genetic resources and precise targets for molecular breeding aimed at improving maize drought tolerance.

## 2. Materials and Methods

### 2.1. Plant Materials and Stress Treatments

The maize inbred line B73 was used as the experimental material [13]. Seeds were hydroponically cultivated in climate-controlled plant growth chambers (BPC500H, Jiupo biotechnology, Fuzhou, China) with a 14 h light/10 h dark photoperiod at 26 °C/22 °C (day/night) until the three-leaf stage. Subsequently, the seedlings were subjected to control conditions (1/2 Hoagland nutrient solution) [14], salt stress (200 mM NaCl solution), or drought stress (20% Polyethylene Glycol6000 solution). To monitor dynamic gene expression, a time-course qRT-PCR analysis was performed at 0, 12, 24, 48, and 72 h (3 days) after PEG or NaCl treatment. At these indicated time points, the roots, stems, and the second fully expanded leaves of seedlings were harvested, immediately frozen in liquid nitrogen, and stored at −80 °C. Each treatment included three biological replicates [14]. For haplotype analysis, 157 waxy maize inbred lines, which were independently bred and purified over many years by the Shanghai Academy of Agricultural Sciences, were used. Population genetic analysis revealed that this population originated from four ancestral subgroups (G1–G4), with the G2 subgroup comprising 143 lines. Seedling drought stress screening was conducted at the Lingshui Experimental Station (18.57° N, 110.09° E) using 128-cell plug trays (3 cm × 3 cm per cell). After irrigation was withheld for 5 days at the two-leaf stage, a drought index (0–5 scale) was scored based on wilting [15,16] (Table 1). Survival was defined as the ability to resume growth (indicated by the emergence of new green leaves or stem re-greening) after re-watering. The survival rate (%) was calculated as (number of surviving plants/total number of plants) × 100% after 3 days of recovery. The experiment was conducted with three biological replicates. Differences in drought index and survival rate among haplotypes were analyzed using ANOVA followed by the least significant difference (LSD) test.

### 2.2. Identification and Chromosomal Distribution Analysis of ZmCAD Genes

First, the maize B73 genome, protein sequences, and GFF3 annotation files were downloaded from the MaizeGDB database (https://maizegdb.org/). To identify *ZmCAD* family members, two complementary strategies were employed: homology-based searching and domain-based searching. For homology-based searching, the amino acid sequences of nine reported Arabidopsis thaliana CAD proteins (AtCAD1-9) were retrieved from the TAIR database (https://www.arabidopsis.org/) [17] and used as query sequences to screen the maize proteome using BLASTP (v2.14.0+) (E-value ≤ 1 × 10^−5^, identity ≥ 30%). For domain-based searching, the Hidden Markov Model (HMM) profile of the CAD-specific conserved domain (PF08240) was obtained from the Pfam database (https://pfam.xfam.org/), and the maize proteome was scanned using HMMER 3.3.2 (E-value ≤ 0.01). Candidate sequences from both approaches were integrated, and their conserved domains were validated using the Pfam (v35.0) and NCBI CDD (v3.20) databases to confirm the final set of members containing a complete CAD domain [18].

### 2.3. Genomic Localization and Physicochemical Property Prediction of ZmCAD Genes

Based on the genomic annotation information, the Gene ID, chromosomal coordinates, and amino acid length of each member were extracted. The molecular weight (MW) and theoretical isoelectric point (pI) were predicted using the ExPASy ProtParam tool (https://web.expasy.org/protparam/, accessed on 8 January 2026) [19]. Finally, a chromosomal physical map was constructed using TBtools software (v1.108) based on the GFF3 file [20] to visualize the distribution of *ZmCAD* members and the gene density on each chromosome.

### 2.4. Analysis of Gene Structure, Conserved Domains, and Motifs of ZmCAD Genes

To analyze the structural features of *ZmCAD* genes, the genomic and CDS sequences of each member were extracted. Conserved protein motifs were predicted using the MEME Suite (v5.5.8) with the following parameters: maximum number of motifs = 10, motif width = 6–100 amino acids, and maximum iterations = 10,000 [21]. The exon-intron structures were visualized using TBtools software (v1.108). Conserved domains were annotated using the NCBI CDD database (E-value < 0.01) [22]. Finally, an integrated diagram illustrating the gene structure, conserved motifs, and domain architecture was generated using TBtools.

### 2.5. Prediction of Secondary, Tertiary Structures, and Subcellular Localization of ZmCAD Proteins

The secondary structures of the ZmCAD proteins were predicted using the SOPMA online tool (https://npsa.lyon.inserm.fr/, accessed on 8 January 2026) [23]. Three-dimensional (3D) structure models were constructed using the SWISS-MODEL online platform (https://swissmodel.expasy.org/interactive, accessed on 8 January 2026) and visualized with PyMOL software (v1.8.6.0) [24]. Structural similarity analysis was performed using the DALI online server (http://ekhidna2.biocenter.helsinki.fi/dali/, accessed on 8 January 2026), and the results were visualized as a heatmap using GraphPad Prism (v9.3.0) [25]. Subcellular localization was predicted using a combination of the Cell-PLoc 2.0 (http://www.csbio.sjtu.edu.cn/bioinf/Cell-PLoc-2/, accessed on 8 January 2026) and WoLF PSORT (https://wolfpsort.hgc.jp/) online tools [26,27].

### 2.6. Phylogenetic Analysis and Tissue Expression Profiles of ZmCAD Genes

To elucidate the phylogenetic relationships of ZmCAD proteins, the amino acid sequences of CAD proteins from Arabidopsis thaliana, rice, and maize were retrieved from the TAIR, NCBI, and MaizeGDB databases. A phylogenetic tree was constructed using the Neighbor-Joining (NJ) method in MEGA-X software (v10.1.8) with the Poisson substitution model. Branch reliability was assessed with 1000 bootstrap replicates. The final tree was visualized and annotated using the iTOL online platform (https://itol.embl.de/, accessed on 8 January 2026). The tissue expression profiles were analyzed using the Zea mays RNA-seq Database (https://plantrnadb.com/zmrna/, accessed on 8 January 2026), wherein the basal expression levels of *ZmCAD* members across different tissues were retrieved from publicly available datasets [28].

### 2.7. Promoter Cis-Element Analysis and Transcription Factor Prediction

The 2.0 kb genomic sequences upstream of the translation start site (ATG) of each *ZmCAD* gene were extracted as putative promoter regions. Cis-acting elements were predicted using the PlantCARE database (http://bioinformatics.psb.ugent.be/webtools/plantcare/html/, accessed on 8 January 2026) and subsequently classified according to their biological functions [29,30]. Transcription factors associated with *ZmCAD* genes were identified via the ZEAMAP platform (https://db.cngb.org/zeamap, accessed on 8 January 2026) [31], and the predictive results were subsequently visualized through Rstudio (v4.5.2).

### 2.8. Cross-Species Ka/Ks and Collinearity Analysis of the ZmCAD Gene Family

To assess the evolutionary selection pressure, orthologous gene pairs of *ZmCADs* among maize, sorghum, and rice were first identified using the MCScanX method (E-value < 1 × 10^−5^, coverage ≥ 70%). Subsequently, the Ka/Ks ratios for both paralogous pairs within maize and orthologous pairs between maize and sorghum were calculated using the Simple Ka/Ks Calculator module in Tbtools [20]. For collinearity analysis, the genomic data of maize, sorghum, and rice were integrated, and MCScanX-transposed was used to identify interspecies collinearity blocks. Finally, the intragenomic collinearity within the maize genome and the interspecies collinearity network among the three species were visualized using the Advanced Circos module in TBtools.

### 2.9. Analysis of Evolutionary Selection Pressure and Functional Network of ZmCAD Genes

To further detect whether the *ZmCAD* gene family underwent positive selection during evolution, the aBSREL (adaptive Branch-Site Random Effects Likelihood) model, available on the Datamonkey online platform (https://www.datamonkey.org/), was employed to analyze the CDS sequences of the nine *ZmCAD* genes. This model identifies sites and branches under positive selection (ω = dN/dS > 1) [32]. To explore the potential biological functions of ZmCAD proteins, a protein–protein interaction (PPI) network was constructed using the STRING database (https://cn.string-db.org/). Subsequently, Gene Ontology (GO) enrichment analysis was performed on the genes in the network to reveal their potential roles in biological processes, molecular functions, and cellular components [33].

### 2.10. Quantitative Real-Time PCR Analysis

To validate the expression patterns of *ZmCAD* genes under stress conditions, qRT-PCR was performed. Reactions were carried out on a QuantStudio™ 6 Flex system (Applied Biosystems, Waltham, MA, USA) using the ChamQ SYBR Color qPCR Master Mix kit (Vazyme, Nanjing, China) [14]. Gene-specific primers were designed using Primer 5.0 (sequences listed in Table S1) and synthesized by Tsingke Biotechnology Co., Ltd. (Shanghai, China). The *ZmUBQ* gene was used as an internal reference. The reaction mixture and thermal cycling program were strictly followed according to the manufacturer’s instructions. Each sample was analyzed with three biological replicates and three technical replicates (with Ct value variation among technical replicates < 0.5). The relative expression levels were calculated using the 2^−ΔΔCT^ method.

### 2.11. Natural Variation and Haplotype Diversity of ZmCAD6

To investigate the natural variation in the *ZmCAD6* gene, the full-length sequences of the *ZmCAD6* gene, including upstream and downstream regulatory regions, were extracted from the genotypic data of 157 waxy maize inbred lines. Following multiple sequence alignment with MAFFT, nucleotide polymorphism and haplotype analyses were conducted using DnaSP (v5.1.0.). The evolutionary relationships among the haplotypes were then visualized in a median-joining network constructed with POPART (v1.7) software [34,35].

## 3. Results

### 3.1. Physicochemical Properties and Chromosomal Distribution of the ZmCADs

Physicochemical analysis revealed significant diversity among the members of the *ZmCAD* gene family. The amino acid (AA) lengths of the ZmCAD proteins ranged from 354 (ZmCAD8) to 432 (ZmCAD1), with corresponding molecular weights (MW) from 37.28 kDa (ZmCAD6) to 47.26 kDa (ZmCAD1). Notably, ZmCAD1 was distinct in size, possessing the highest AA count (432) and MW (47.26 kDa), whereas the other eight members exhibited relatively conserved dimensions (354–414 AA; 37.28–43.46 kDa). The theoretical isoelectric points (pI) of the ZmCAD proteins ranged from 5.52 (ZmCAD5) to 8.62 (ZmCAD1). ZmCAD1 was the sole member predicted to be alkaline (pI > 7.0), while the remaining proteins (ZmCAD2–ZmCAD9) were acidic or neutral (pI: 5.52–7.19). The instability indices for all members were below 40, indicating they are stable proteins. Among them, ZmCAD4 was the most stable (instability index = 22.39), whereas ZmCAD1 was the least stable (instability index = 37.65). Hydrophobicity analysis further highlighted differences among the family members. ZmCAD4 exhibited the highest hydrophobicity, with an aliphatic index of 96.1 and a GRAVY value of 0.162. In contrast, ZmCAD1 was predicted to be relatively hydrophilic (GRAVY = −0.17). A significant distinction was observed in subcellular localization predictions: ZmCAD1 was the only member predicted to localize to the mitochondrion, whereas all other members were predicted to be cytoplasmic. In summary, the ZmCAD family members exhibit considerable physicochemical divergence. The unique combination of a large size, alkaline nature, and mitochondrial localization distinguishes ZmCAD1 from the other family members, which are generally smaller, acidic/neutral, and cytoplasmic (Table 2).

**Table 2:** Predicted physicochemical properties and subcellular localization of ZmCAD proteins.

Protein	AA Count	MW (kDa)	pI	Instab. Index	Aliph. Index	GRAVY	Localization
ZmCAD1	432	47.26	8.62	37.65	81.67	−0.17	Mitochondrial
ZmCAD2	370	39.48	6.31	34.36	85.43	−0.037	Cytoplasm
ZmCAD3	361	38.71	6.82	27.51	92.33	0.048	Cytoplasm
ZmCAD4	367	38.73	5.95	22.39	96.1	0.162	Cytoplasm
ZmCAD5	365	37.96	5.52	26.44	88.08	0.152	Cytoplasm
ZmCAD6	358	37.28	6.11	23.07	89.47	0.115	Cytoplasm
ZmCAD7	414	43.46	7.19	32.1	86.62	0.028	Cytoplasm
ZmCAD8	354	38.38	6.82	25.34	88.36	−0.018	Cytoplasm
ZmCAD9	359	38.00	6.45	29.57	86.38	0.061	Cytoplasm

**Figure 1:**
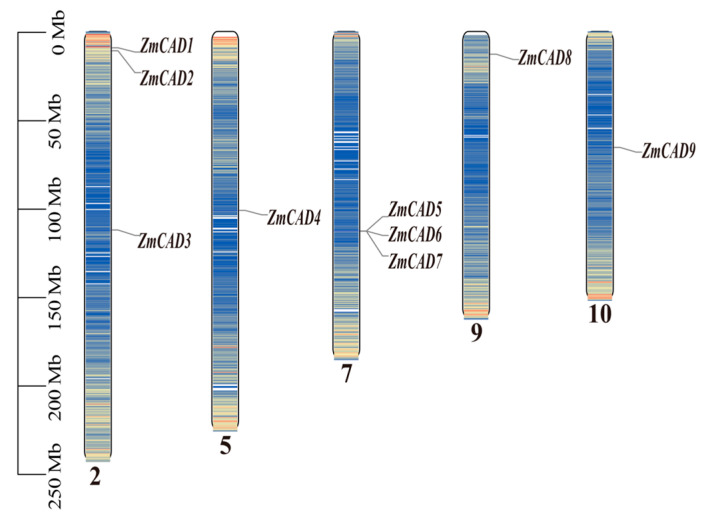
**Physical localization of *ZmCAD* gene family members on chromosomes in maize.** The figure shows the distribution of *ZmCAD* gene family members on the 10 maize chromosomes. The scale on the left indicates the physical length of the chromosomes (in Mb). The Arabic numerals (2, 5, 7, 9, 10) below the chromosomes represent the chromosome numbers, and the gene names are marked at their respective positions.

In addition, the nine *ZmCAD* genes were distributed across five maize chromosomes (2, 5, 7, 9, and 10). Specifically, three genes (*ZmCAD1*, *ZmCAD2*, and *ZmCAD3*) were located on chromosome 2, and another three (*ZmCAD5*, *ZmCAD6*, and *ZmCAD7*) were found on chromosome 7. The remaining three genes, *ZmCAD4*, *ZmCAD8*, and *ZmCAD9*, were each located on chromosomes 5, 9, and 10, respectively. Notably, *ZmCAD5*, *ZmCAD6*, and *ZmCAD7* formed a physical cluster on chromosome 7. Overall, the distribution of the *ZmCAD* genes did not follow a discernible pattern, with some members located in pericentromeric regions and others in distal or telomeric regions (Figure 1).

### 3.2. Gene Structure and Protein Architecture of the ZmCADs

**Figure 2:**
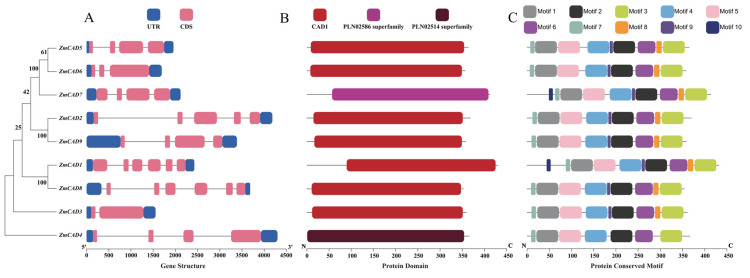
Analysis of gene structure and protein architecture for the *ZmCAD* gene family. (**A**) Schematic representation of the gene structures for the nine *ZmCAD* genes, illustrating their exon-intron organization, including 5′ and 3′ untranslated regions (UTRs). (**B**) Conserved domain composition of the ZmCAD proteins. (**C**) Conserved motif composition of the ZmCAD proteins. Different colors are used to distinguish different structural elements.

Gene structure analysis revealed that all nine *ZmCAD* genes possess 5′ and 3′ untranslated regions (UTRs) and a multi-exon and intron structure. The number of exons and introns varied among the members. *ZmCAD8* contained the highest number of both exons and introns (eight each), whereas *ZmCAD1* had the fewest introns (one) and *ZmCAD6* had the fewest exons (three) (Figure 2A). In terms of protein domain composition, with the exception of ZmCAD7, which possessed the PLN02586 superfamily domain, and ZmCAD4, which had the PLN02514 superfamily domain, all other ZmCAD proteins contained the typical CAD1 (cd05283) domain. All three identified domain types are associated with cinnamyl alcohol dehydrogenase (Figure 2B). Furthermore, an analysis of conserved motifs identified a total of 10 distinct motifs across the nine ZmCAD proteins. Notably, only ZmCAD1 and ZmCAD7 contained all 10 motifs, while the remaining members lacked motif 10. This variation in motif composition suggests potential functional divergence within the gene family (Figure 2C).

### 3.3. Secondary and Tertiary Structure Prediction of ZmCAD Proteins

**Figure 3:**
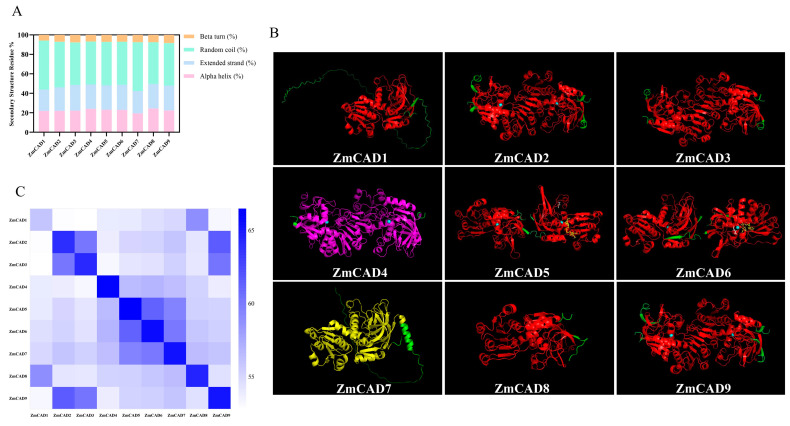
**Secondary and tertiary structure analysis of ZmCAD proteins.** (**A**) The secondary structure composition of the ZmCAD proteins. (**B**) Predicted three-dimensional (3D) structures of the ZmCAD proteins. The CAD1 domain is shown in red, the PLN02586 superfamily domain in yellow, the PLN02514 superfamily domain in purple, zinc ions as blue spheres, and linker peptides in green. (**C**) Heatmap illustrating the 3D structural similarity among ZmCAD proteins. The color gradient from light to dark blue indicates increasing similarity.

**Figure 4:**
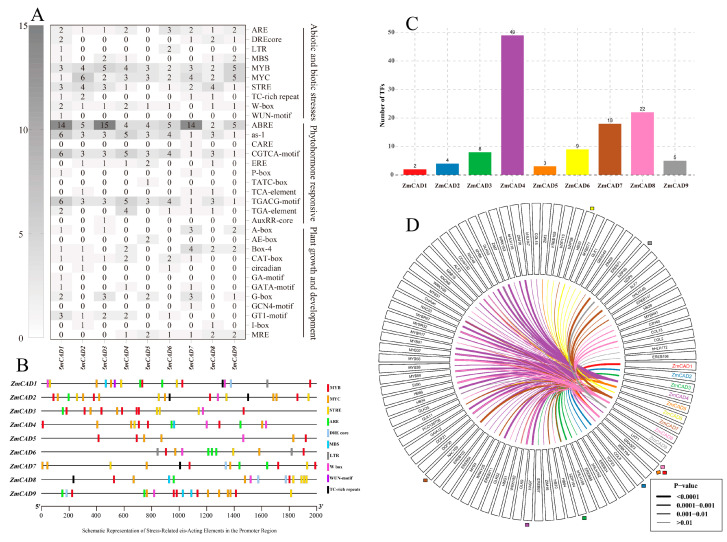
**Promoter cis-element analysis and transcription factor prediction of the *ZmCAD* gene family.** (**A**) Functional classification and quantitative statistics of cis-acting elements in the promoters of the *ZmCAD* gene family. These are classified into three major functional categories: stress response, hormone response, and growth and development regulation. (**B**) Schematic diagram of the number and distribution of stress-responsive cis-acting elements in the promoters of *ZmCAD* gene family members. Different colored boxes in the figure represent different cis-acting elements. (**C**) Comparison of the number of predicted binding transcription factors for different *ZmCAD* genes. (**D**) Detailed visualization of the predicted transcription factors binding to *ZmCAD* genes. Distinct colors represent individual *ZmCAD* genes. The colored squares surrounding the circle denote the transcription factors with the highest prediction confidence for binding to each corresponding gene.

Analysis of the secondary structure composition revealed that random coils were the predominant component in all ZmCAD proteins, ranging from 43.33% (ZmCAD9) to 50.46% (ZmCAD1). The proportion of extended strands varied considerably, from 21.99% (ZmCAD1) to 26.39% (ZmCAD3), with ZmCAD3 showing the highest content. The α-helix content ranged from 19.32% (ZmCAD7) to 24.43% (ZmCAD8), while the β-turn content was relatively conserved (5.79–8.33%) (Figure 3A). To further investigate the three-dimensional (3D) conformation, homology modeling was performed using Swiss-Model. The predictions indicated that ZmCAD1, ZmCAD7, and ZmCAD8 adopt a monomeric conformation, whereas the other six members (ZmCAD2, ZmCAD4-ZmCAD6, and ZmCAD9) form homodimers (Figure 3B). Further analysis of the dimeric proteins revealed the presence of a predicted zinc ion-binding site at the N-terminus in all members except ZmCAD3 (Figure 4).

Finally, a heatmap of 3D structural similarity was constructed to compare the predicted models (Figure 3C). The analysis showed the highest similarity score between ZmCAD2 and ZmCAD9, indicating a highly conserved 3D conformation. Additionally, significant structural similarities were also observed between ZmCAD9 and ZmCAD3, as well as between ZmCAD1 and ZmCAD8.

### 3.4. Promoter Analysis of the ZmCAD Gene Family

To investigate the cis-regulatory features of the *ZmCAD* gene family, the 2.0 kb upstream promoter regions of each gene were analyzed. Cis-acting elements were annotated using the PlantCARE database and classified into three major functional categories: stress response, hormone response, and growth and development regulation (Figure 4A). In total, 33 distinct cis-acting elements were identified, including 10 related to stress, 11 to hormones, and 12 to development. Among the stress-responsive elements, MYB and MYC binding sites were ubiquitous across all family members. The antioxidant response element (ARE) and stress-responsive element (STRE) were the most frequent abiotic stress-related elements. Notably, the wound-responsive element (WUN-motif) was uniquely present in the promoter of *ZmCAD1*. Furthermore, *ZmCAD5* contained the fewest stress-related elements (7), which was significantly lower than the other members (11–18) (Figure 4B). For hormone-responsive elements, the abscisic acid-responsive element (ABRE) was the most abundant, with more than 14 copies detected in the promoters of *ZmCAD1*, *ZmCAD3*, and *ZmCAD7*. This was followed by the methyl jasmonate (CGTCA-motif) and gibberellin (TGACG-motif) responsive elements. In contrast, the auxin-responsive element (AuxRR-core) was detected only in *ZmCAD3*. Although the total number of growth and development-related elements was the lowest among the three categories, *ZmCAD7* was a distinct exception, possessing 14 such elements, considerably more than the other members (4–10). These distinct patterns in cis-element composition and distribution provide regulatory-level evidence for the functional divergence of the *ZmCAD* gene family. Transcription factor prediction results revealed significant differences in the number of binding transcription factors among different *ZmCAD* gene family members. Specifically, *ZmCAD4* exhibited the highest number of predicted binding transcription factors, reaching 49, whereas *ZmCAD1* and *ZmCAD5* were associated with only 2–3. *ZmCAD7* and *ZmCAD8* also presented relatively high binding counts, both exceeding 10, while *ZmCAD6* and *ZmCAD3* displayed moderate levels with 9 and 8 binding factors, respectively. Visualization analysis of the predicted binding transcription factors across the nine *ZmCAD* members revealed that GRAS23 is the shared transcription factor with the highest prediction confidence for *ZmCAD1*, *ZmCAD5*, and *ZmCAD8*. Furthermore, the transcription factors predicted to bind to *ZmCAD* genes were primarily classified into the MYB, WRKY, bZIP, and EREB families, most of which are closely associated with plant stress responses.

### 3.5. Evolutionary Analysis of the ZmCAD Gene Family

**Figure 5:**
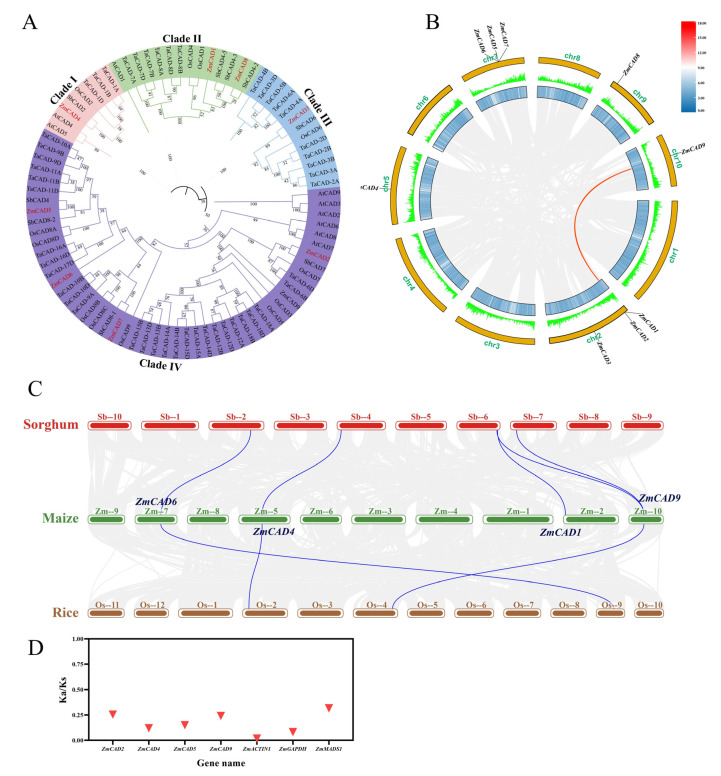
**Phylogenetic and collinearity analysis of the *ZmCAD* gene family.** (**A**) Phylogenetic tree constructed using CAD protein sequences from maize (*Zea mays*), Arabidopsis (*Arabidopsis thaliana*), wheat (*Triticum aestivum*), rice (*Oryza sativa*), and sorghum (*Sorghum bicolor*). In the tree, ZmCAD, OsCAD, TaCAD, AtCAD, and SbCAD represent CAD proteins from maize, rice, wheat, Arabidopsis, and sorghum, respectively. (**B**) Intraspecific collinearity analysis within the maize genome. (**C**) Interspecific collinearity analysis among maize, sorghum, and rice genomes. (**D**) Analysis of Ka/Ks values.

To elucidate the evolutionary relationships within the *CAD* gene family, a phylogenetic tree was constructed using CAD protein sequences from maize, Arabidopsis, wheat, rice, and sorghum (Figure 5A). The nine ZmCAD proteins were classified into four distinct clades. ZmCAD2, ZmCAD5, ZmCAD6, and ZmCAD7 clustered in Clade VI; ZmCAD8 and ZmCAD9 were placed in Clade II; while ZmCAD3 and ZmCAD4 were located in Clade III and Clade I, respectively.

Interspecies collinearity analysis among maize, sorghum, and rice revealed conserved orthologous relationships within these grass species (Figure 5C). Specifically, four orthologous gene pairs were identified between maize and sorghum (*ZmCAD1*, *ZmCAD4*, *ZmCAD6*, *ZmCAD9*), and three between maize and rice (*ZmCAD4*, *ZmCAD6*, *ZmCAD9*). Intraspecies analysis within maize identified only one paralogous gene pair involving *ZmCAD9* (Figure 5B). To assess the selective pressures acting on these genes, the Ka/Ks ratios of the orthologous gene pairs were calculated (Figure 5D). The Ka/Ks values for *ZmCAD*1, 4, 6, and 9 were all significantly less than 1, indicating that these genes have undergone strong purifying selection. For context, their Ka/Ks values were comparable to the housekeeping gene *ZmGAPDH* and lower than the developmental gene *ZmMADS1*, with *ZmCAD4* exhibiting a value particularly close to that of *ZmGAPDH*.

Furthermore, branch-site model analysis was performed to detect signatures of positive selection across the phylogeny. A significant signal of positive selection was detected only on the evolutionary branch leading to *ZmCAD7* (Table S2).

### 3.6. Protein–Protein Interaction Network Analysis of ZmCADs

**Figure 6:**
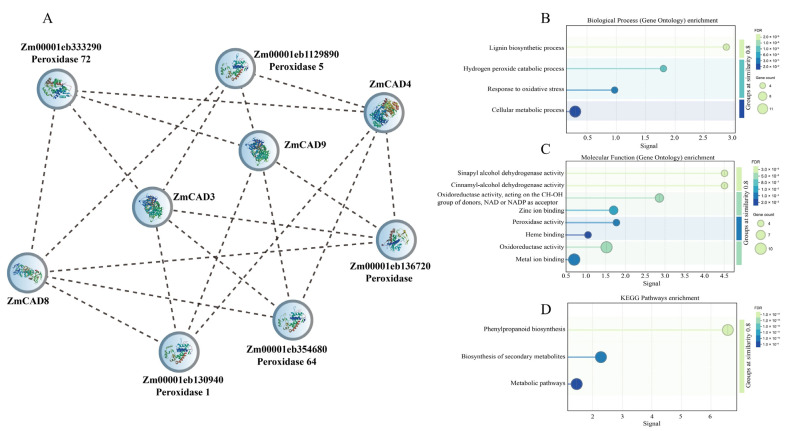
**Analysis of the PPI network and functional enrichment for the core ZmCAD module.** (**A**) The PPI network of the core module, comprising ZmCAD3, ZmCAD4, ZmCAD8, and ZmCAD9. (**B**) GO enrichment analysis for Biological Processes. (**C**) GO enrichment analysis for Molecular Functions. (**D**) KEGG pathway enrichment analysis.

PPI network analysis identified four primary interaction modules. A major module comprised ZmCAD3, ZmCAD4, ZmCAD8, and ZmCAD9, which interacted with five peroxidase proteins (Figure 6A). GO enrichment analysis for this module showed significant enrichment in biological processes such as the lignin biosynthetic process, hydrogen peroxide catabolic process, response to oxidative stress, and cellular metabolic processes (Figure 6B). The molecular functions of the interacting proteins were predominantly associated with peroxidase activity and oxidoreductase activity (Figure 6C). Furthermore, KEGG pathway analysis identified phenylpropanoid biosynthesis as the only significantly enriched pathway (Figure 6D).

The interaction network for ZmCAD1 was distinct, with its interacting proteins significantly enriched in RNA metabolic processes (e.g., RNA processing, degradation) and gene expression regulation (Figure S1A). The corresponding molecular functions were enriched for RNA binding (Figure S1B), and KEGG analysis highlighted the ribosome biogenesis pathway in eukaryotes.

Finally, ZmCAD6 was predicted to interact with proteins including Fasciclin-like arabinogalactan proteins 6 and 7 (FLA6, FLA7), Expansin-like 3 (EXPL3), and Caffeoyl-CoA O-methyltransferase 1 (CCoAOMT1) (Figure S1H).

### 3.7. Tissue-Specific and Stress-Responsive Expression Profiles of ZmCADs

Based on the analysis of 110 transcriptomic datasets, we characterized the expression profiles of nine *CAD* gene members across various maize tissues. The results revealed distinct expression patterns among these genes. While the majority of members exhibited moderate to high expression levels in major vegetative tissues such as roots, stems, and leaves, *ZmCAD2* and *ZmCAD8* showed low expression in roots, and *ZmCAD1* and *ZmCAD2* were lowly expressed in stems and leaves. Notably, *ZmCAD1* and *ZmCAD2* maintained high or moderate expression levels in reproductive tissues including seeds, endosperm, silks, and anthers, whereas other members were significantly lowly expressed in these tissues. In seedlings, *ZmCAD1* and *ZmCAD9* displayed relatively low expression, while the remaining members, particularly *ZmCAD6* and *ZmCAD7*, exhibited significantly high expression levels.

**Figure 7:**
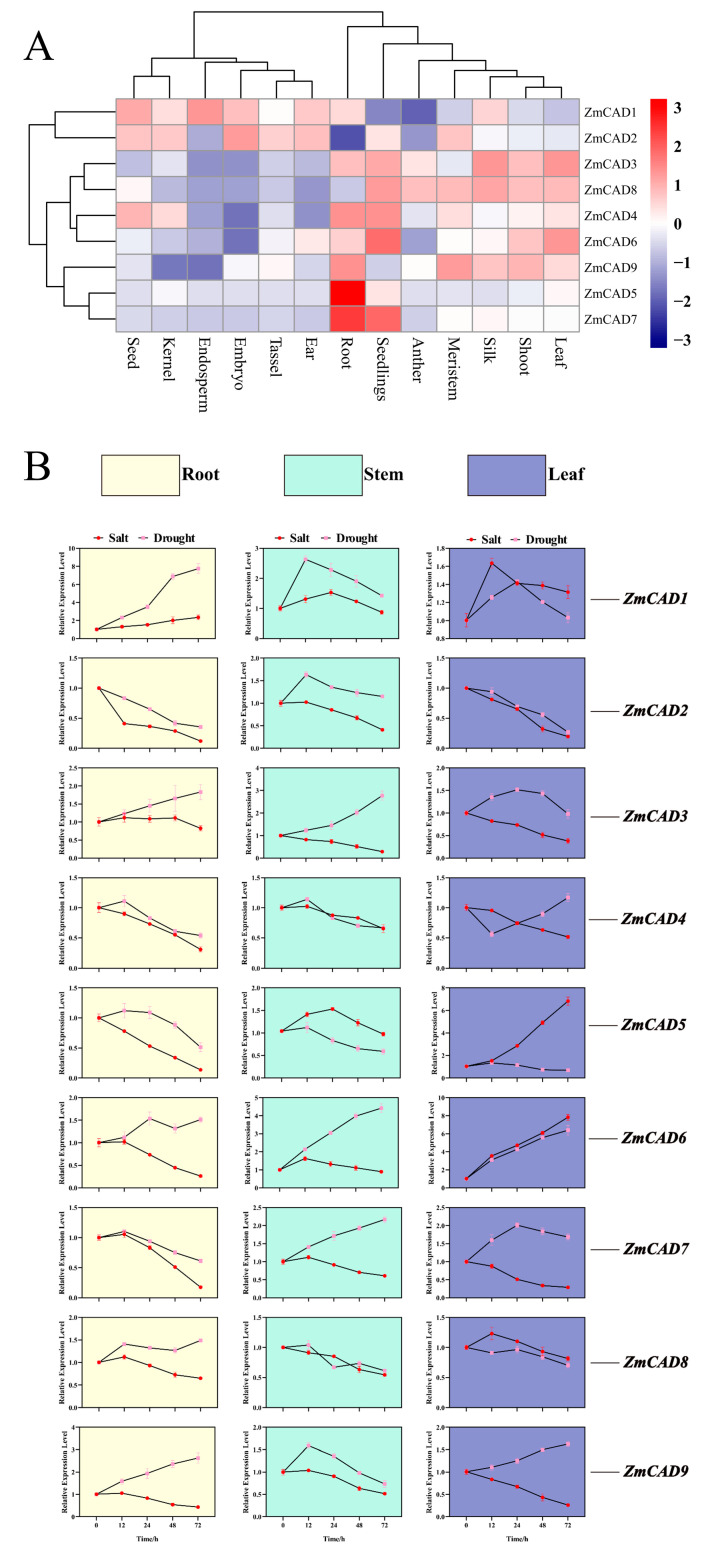
**Expression analysis of *ZmCAD* genes.** (**A**) Tissue-specific expression profiles of *ZmCAD* genes shown as a heatmap. Values were Z-score normalized based on FPKM. Red and blue indicate high and low expression, respectively, while white represents moderate expression. (**B**) Tissue types and stress treatments are indicated by color: yellow (root), cyan (stem), blue (leaf), red (salt stress), and pink (drought stress).

To elucidate the response patterns of the *ZmCAD* gene family under abiotic stress, we analyzed the relative expression levels of nine *ZmCAD* members in the roots, stems, and leaves of maize seedlings after 3 days of treatment with 200 mM NaCl (salt stress) and 20% PEG6000 (simulated drought stress) using qRT-PCR, with *ZmUBQ* as the reference gene (Figure 7B). The results revealed that the expression patterns of family members were complex and highly tissue-specific.

Notably, *ZmCAD6* exhibited a unique and consistent response pattern, suggesting its pivotal role in drought response. Under drought stress, its expression was significantly upregulated in all three tissues, with the most pronounced increase observed in leaves (approximately 6-fold). In contrast, its upregulation under salt stress was largely confined to leaf tissue, indicating that *ZmCAD6* may play a key role in the maize response to drought.

Other members also displayed diverse regulatory patterns, reflecting functional differentiation within the family. For instance, *ZmCAD3* and *ZmCAD9* showed a conserved trend of being “drought-induced and salt-repressed” across all tissues. *ZmCAD1* was consistently upregulated under both stresses in all tissues, with an increase of up to approximately 8-fold in roots under drought stress. Conversely, *ZmCAD2* and *ZmCAD8* were generally downregulated under most conditions, with *ZmCAD2* being strongly suppressed in leaves under salt stress. Furthermore, the expression patterns of *ZmCAD4*, *ZmCAD5*, and *ZmCAD7* were more complex, with their responses varying significantly among tissues even under the same stress. Collectively, these results suggest that members of the *ZmCAD* gene family collectively regulate the complex response network of maize to abiotic stresses through functional differentiation and synergistic effects.

### 3.8. Identification of an Elite Drought-Tolerant Haplotype of ZmCAD6

**Figure 8:**
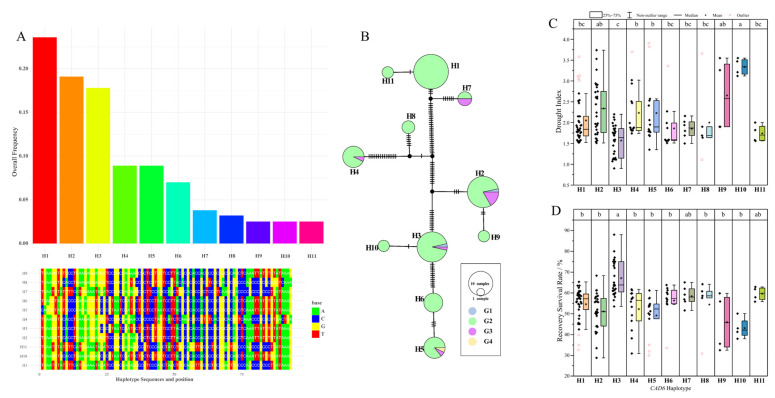
**Association analysis of *ZmCAD6* natural variation with drought tolerance.** (**A**) Sequence polymorphisms and distribution of 11 *ZmCAD6* haplotypes (H1-H11) identified in a population of 157 waxy maize inbred lines. The analysis was based on a 93 bp polymorphic sequence. (**B**) Median-joining network illustrating the genetic relationships and population structure of the 11 haplotypes. The size of each circle is proportional to the number of inbred lines carrying that haplotype, and different colors indicate distinct genetic subpopulations. (**C**) Box plot showing the average drought index for inbred lines carrying different *ZmCAD6* haplotypes under drought stress. A lower index indicates higher drought tolerance. (**D**) Box plot showing the average survival rate after re-watering for inbred lines carrying different *ZmCAD6* haplotypes. In panels (**C**,**D**), values represented by bars with different lowercase letters are significantly different at the *p* < 0.05 level.

To explore the potential link between natural variation in the *ZmCAD6* gene and drought tolerance in maize, we conducted a comprehensive haplotype analysis using a panel of 157 waxy maize inbred lines. The *ZmCAD6* gene was universally present across all tested accessions and showed an extremely low level of nucleotide diversity (Pi = 0.00782), indicating that this gene has undergone strong purifying selection during its evolutionary history. Based on sequence variation within a 93 bp region, we identified a total of 11 distinct haplotypes, designated H1 through H11 (Figure 8A). Among these, H1, H2, and H3 were the three most prevalent haplotypes, found in 37, 30, and 28 inbred lines, respectively. To further understand the population structure, a median-joining network was constructed (Figure 8B). The network analysis revealed that haplotypes H3, H2, and H5 were broadly distributed across three different genetic subpopulations, while H1 was specifically confined to subpopulation II. A subsequent association analysis was performed to link haplotypes to drought tolerance phenotypes, uncovering a significant correlation (Figure 8C). Inbred lines carrying the H9 and H10 haplotypes demonstrated the highest average drought index, suggesting they were the most susceptible to drought stress. In stark contrast, lines harboring the H3 haplotype exhibited the most robust drought tolerance, with an average drought index of merely 1.57, a value significantly lower than that of all other haplotypes. This superior performance was further corroborated by the survival rate after re-watering; the H3 group achieved an average survival rate of 67.1%, representing an approximate increase of 13% and 16% compared to the numerically comparable H1 and H2 haplotype groups, respectively. Taken together, these findings robustly identify the H3 haplotype of *ZmCAD6* as an elite allele strongly associated with enhanced drought tolerance in maize, thus providing a valuable genetic resource and a promising molecular marker for future drought-resistance breeding programs.

## 4. Discussion

Abiotic stresses, particularly drought and salinization, are critical environmental factors limiting maize yield. Lignin, as a key cell wall component, plays an indispensable role in enhancing plant mechanical strength and resisting adverse stresses. Cinnamyl alcohol dehydrogenase is a terminal key enzyme in the lignin biosynthetic pathway. While its function has been closely linked to both biotic and abiotic stress responses in various species—for instance, overexpression of CAD genes in soybean and cotton enhances disease resistance [11,36,37]—its systematic functional analysis in response to abiotic stress in maize, particularly its potential for application in stress-resistant genetic improvement, remains elusive. This study presents the first comprehensive, multi-dimensional analysis of the maize *ZmCAD* gene family, yielding a pivotal finding: we not only reveal the functional differentiation of family members in response to abiotic stress but, more importantly, identify an elite haplotype (H3) of the *ZmCAD6* gene that is significantly associated with strong drought tolerance in maize, providing a valuable genetic resource for molecular breeding.

Our results provide robust evolutionary and structural evidence for the functional differentiation within the *ZmCAD* family. Phylogenetic and collinearity analyses indicate that *ZmCAD5*, *ZmCAD6*, and *ZmCAD7* originated from a recent tandem duplication event. Among them, ZmCAD6, as the ancestral gene, has been subjected to strong purifying selection and remains functionally conserved, whereas *ZmCAD7* underwent positive selection, driving its functional evolution. This functional differentiation is not an isolated case within the family but a widespread phenomenon. A more extreme example is *ZmCAD1*, bioinformatic predictions indicate that *ZmCAD1* possesses unique physicochemical properties, a putative mitochondrial sub-cellular localization, and a predicted protein–protein interaction network related to RNA metabolism. These features collectively suggest a potential biological function distinct from other cytoplasmic *ZmCAD* members, potentially involving organelle-specific metabolic regulation. In contrast, the differentiation of *ZmCAD5/6/7* is more reflected in tissue specificity and stress responses; for example, the predicted interaction of *ZmCAD6* with cell wall assembly proteins implies a potential core role in foundational lignification, while the preferential expression of *ZmCAD7* in leaves implies its functional specialization. Furthermore, the potential association of *ZmCAD2* with the terpenoid synthesis pathway further broadens the possible metabolic networks involving the CAD family, indicating that its functions extend far beyond lignin synthesis.

This evolutionary divergence is ultimately manifested in the stress response patterns. Our expression analysis results are consistent with the above functional predictions. A prime example is *ZmCAD6,* which, despite its high sequence similarity to *ZmCAD5*, exhibits a distinct stress response logic: under drought; it is upregulated in all three tissues, with the most pronounced induction in leaves, a response may be closely related to its function of reinforcing vascular bundles to reduce water loss. Conversely, *ZmCAD3* and *ZmCAD9* display a conserved “drought-induced, salt-repressed” pattern across all tissues. This diametrically opposite regulatory pattern may reflect the plant’s survival strategy trade-offs under different stresses: promoting lignification to conserve water under drought, whereas under salt stress, plants may prioritize limited metabolic resources for ion homeostasis and osmotic adjustment, inhibiting the expression of some *CAD* genes potentially as a resource reallocation strategy to balance growth and survival [38].

Among the numerous candidate genes, *ZmCAD6* stood out due to its unique drought response profile. To investigate whether its natural variation is directly associated with phenotype, we conducted a haplotype analysis, which yielded the most groundbreaking finding of this study. We found that while *ZmCAD6* is highly conserved within the natural population, 11 haplotypes were identified. Crucially, a significant association was found between these haplotypes and drought tolerance phenotypes: inbred lines carrying the H3 haplotype exhibited the lowest drought index and highest post-rehydration survival rate, significantly outperforming other haplotypes. The broad distribution of the H3 haplotype across diverse genetic backgrounds further confirms its stability and broad applicability as an elite allele. This finding successfully establishes a direct link between a stress-responsive gene and an elite haplotype with practical breeding applications, representing a significant leap from “gene function” to “breeding application” and providing an ideal target for molecular marker-assisted selection.

In summary, this study systematically analyzed the evolution, structure, and function of the maize *ZmCAD* gene family, revealing its complex regulatory network in response to abiotic stress. We not only elucidated the mechanisms of functional differentiation among family members but, more importantly, identified and validated the core value of the elite haplotype ZmCAD6-H3 in maize drought tolerance. Of course, this study also has certain limitations. For instance, the identification of the elite haplotype H3 was based on a waxy maize population, and its effects in a broader range of maize germplasm require further validation. Furthermore, the specific molecular mechanism by which ZmCAD6-H3 enhances drought tolerance remains to be elucidated. Future research will focus on utilizing gene editing (e.g., CRISPR/Cas9) or overexpression techniques to functionally validate the predicted roles of *ZmCAD6* and to deeply investigate how the H3 haplotype influences gene expression or enzyme activity, thereby providing a more explicit theoretical basis and efficient genetic tools for the precise improvement of drought tolerance in maize, holding both significant theoretical importance and promising application prospects.

## 5. Conclusions

In this study, we conducted the first comprehensive, multi-dimensional analysis of the maize *ZmCAD* gene family, identifying nine members that exhibit significant divergence in gene structure, evolutionary relationships, and protein properties. Expression profiling revealed that *ZmCAD* genes possess complex, tissue-specific responses to abiotic stress, with *ZmCAD6* demonstrating a consistent and strong induction under drought conditions. Furthermore, haplotype analysis of a waxy maize population successfully identified an elite haplotype, H3, of *ZmCAD6*. This haplotype is significantly associated with superior drought tolerance, characterized by a lower drought index and higher survival rates after re-watering. Collectively, these findings not only elucidate the functional landscape of the *CAD* family in maize but also highlight the ZmCAD6-H3 haplotype as a valuable genetic resource and a precise molecular target for future molecular breeding programs aimed at enhancing drought tolerance in maize.

## Figures and Tables

**Table 1:** Scoring criteria for drought stress evaluation.

Drought Index	Phenotypic Description
0	Normal growth, fully expanded leaves, no wilting symptoms.
1	Several leaves partially rolled and yellowed; plant erect but leaves flaccid.
2	Majority of leaves rolled into a tubular shape but still turgid; plant begins to lean.
3	All leaves severely rolled into a needle-like shape; stems softened; plant is prostrate/lodged.
4	Plant near death; leaves and stems are desiccated and bleached, but with signs of life.
5	Plant completely dead and fully desiccated.

## Data Availability

The original contributions presented in this study are included in the article/Supplementary Materials. Further inquiries can be directed to the corresponding authors.

## Supplementary Materials

The following supporting information can be downloaded at: https://www.mdpi.com/article/10.3390/plants15020241/s1, Figure S1: Protein–protein interaction network and functional enrichment analysis of *ZmCAD1/2/5*; Table S1: Primers and their sequences used in this study. Table S2: Results of the branch-site model analysis for the *ZmCAD* gene family; Table S3: The amino acid sequences for motifs 1–10 of the ZmCAD family identified by MEME analysis; Table S4: The CAD protein sequences used to construct the phylogenetic tree in Figure 5A. Table S5: Information on *ZmCAD6* haplotypes and ancestral populations of the waxy maize inbred lines.
