# Supplementary figures and images for: Systematic Analysis of the Maize CAD Gene Family and Identification of an Elite Drought-Tolerant Haplotype of ZmCAD6
Source: Plants (Basel). 2026 Jan 13;15(2):241. doi: 10.3390/plants15020241 (PMC12845355; doi:10.3390/plants15020241)

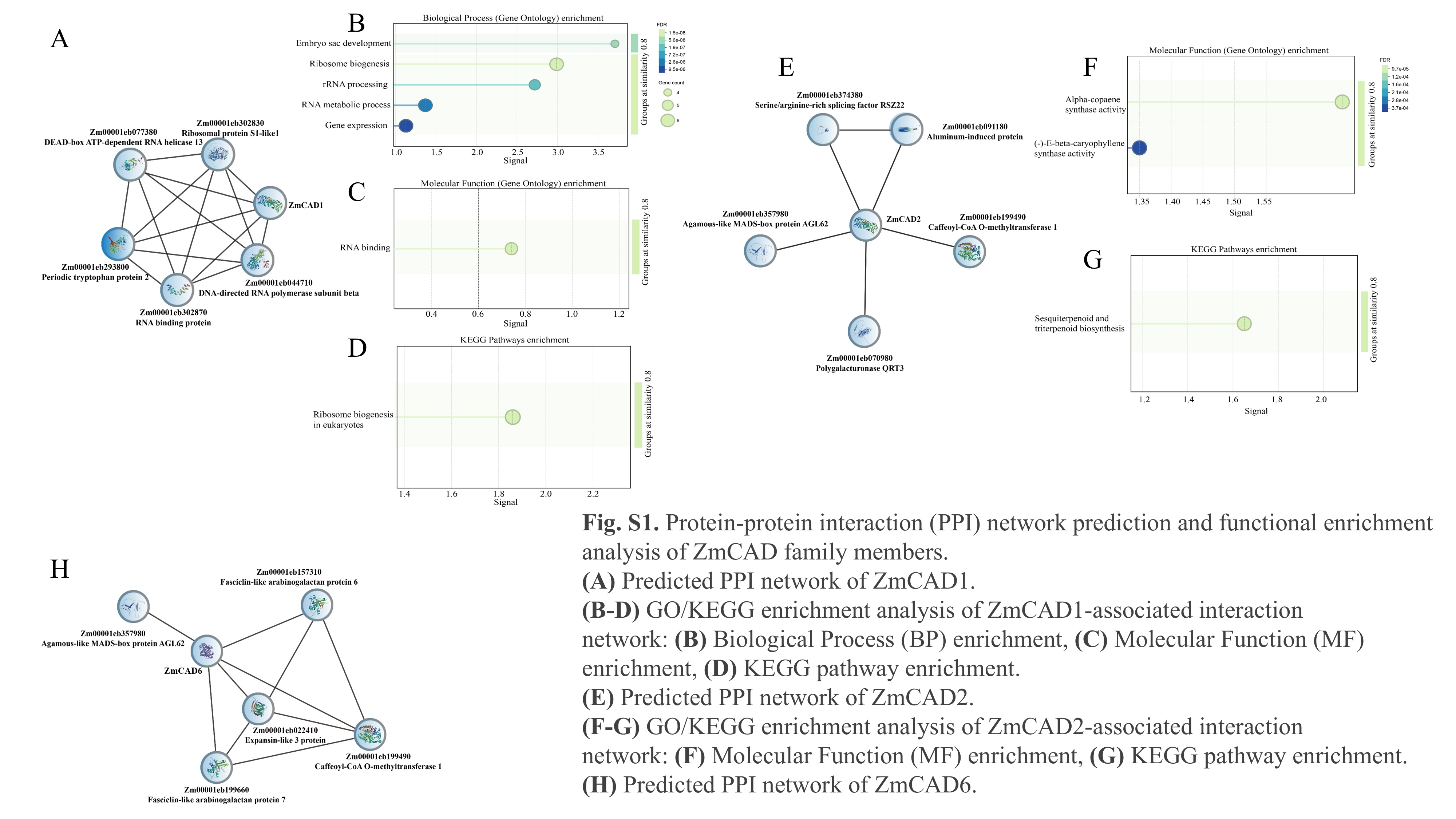

Supplement: Supplementary file 1 [file plants-15-00241-s001.zip › Figure S1.png]
